# Computer Vision in Upper Limb Orthopaedics: A Scoping Review of Imaging-Based Algorithms for Fracture Detection and Radiographic Measurement

**DOI:** 10.7759/cureus.99729

**Published:** 2025-12-20

**Authors:** Nimra Akram, Bilal Qaddoura, Donia Karimaghaei, Sirtaaj Mattoo, Abdullah Al-Jumaili, Sarkhell Radha

**Affiliations:** 1 Trauma and Orthopaedics, Royal Berkshire NHS Foundation Trust, Reading, GBR; 2 Trauma and Orthopaedics, University Hospital Southampton NHS Foundation Trust, Southampton, GBR; 3 Trauma and Orthopaedics, Epsom and St Helier University Hospitals NHS Trust, London, GBR; 4 Trauma and Orthopaedics, Croydon University Hospital, London, GBR; 5 Orthopaedics, Croydon University Hospital, London, GBR

**Keywords:** artificial intelligence (ai), computer vision, msk radiology, orthopaedic surgery shoulder and elbow and upper extremity, scoping review

## Abstract

Computer vision techniques are increasingly applied to medical imaging and may provide valuable assistance in upper limb orthopaedics, a field where radiographs, CT, MRI, and ultrasound are central to diagnosis and treatment planning. The development of deep learning has made automated interpretation of orthopaedic imaging both feasible and increasingly accurate. This review maps and characterises the current use of computer vision in upper limb orthopaedics, describing the clinical problems addressed, the imaging modalities used, and the methodological approaches reported in the literature.

Ovid MEDLINE and Embase were searched from January 1995 to October 2025. Studies were eligible if they applied an automated or semi-automated computer vision technique to upper limb imaging for diagnostic or planning purposes. Conference abstracts and non-orthopaedic applications were excluded. Two reviewers independently screened titles, abstracts, and full texts. Data extraction followed the PRISMA extension for scoping reviews (PRISMA-ScR) framework.

Sixteen studies met the inclusion criteria. Most focused on wrist and shoulder imaging, particularly radiograph-based fracture detection and postoperative morphometric assessment. Convolutional neural networks, detection networks (e.g., YOLO), and segmentation architectures (e.g., U-Net) were most commonly used. Performance was generally high across fracture classification and measurement tasks, although most studies were retrospective, single-centre-based, and lacked external validation.

Computer vision in upper limb orthopaedics remains at an early but promising stage. Automated systems for fracture detection and radiographic measurement demonstrate encouraging accuracy, yet widespread clinical use is limited by small datasets, lack of prospective validation, and inconsistent reporting. Future research should prioritise reproducible multicentre studies, inclusion of soft-tissue modalities, and exploration of intra-operative and real-time clinical applications.

## Introduction and background

Imaging is central to the diagnosis, treatment planning, and postoperative assessment of upper limb conditions. From subtle wrist fractures to complex shoulder arthroplasties, radiographs, CT, MRI, and ultrasound support the orthopaedic decision-making process. However, interpretation of these images is often subjective and dependent on the clinician’s experience. Inter-observer variability and the increasing volume of imaging performed each day have prompted a search for automated methods that can assist clinicians with image interpretation [1]. The clinical and economic burden of upper limb pathology is substantial. Acute trauma, such as distal radius and scaphoid fractures, represents one of the most common workloads in emergency departments. Chronic conditions, like rotator cuff tendinopathy and shoulder osteoarthritis, are primary drivers of disability and surgical intervention in the ageing population [2]. Taken together, these acute and chronic presentations illustrate a large pool of imaging-dependent decisions in which computer vision tools could reduce diagnostic error and standardise reporting. 

This high volume creates a significant diagnostic challenge. The manual interpretation of upper limb imaging is fraught with well-documented pitfalls. Missed scaphoid fractures on initial radiographs, for example, are an infamous source of litigation, as a missed diagnosis can lead to complications like avascular necrosis and non-union. In the shoulder, classifying complex proximal humerus fractures is highly subjective, yet the classification often dictates the choice between non-operative management, internal fixation, or arthroplasty [3]. These examples highlight how variability and misinterpretation can directly translate into delayed diagnosis and suboptimal treatment decisions. Several of the models identified in this review are explicitly designed to address these issues by automatically flagging likely fractures or providing more consistent fracture classification, offering a plausible route to fewer missed injuries and more reproducible management choices.

In elective surgery, planning relies on precise radiographic measurements that are tedious and variable. For shoulder arthroplasty, restoring the patient's native anatomy requires accurate measurement of glenoid version, inclination, and humeral offset. Miscalculation of these parameters can lead to component malpositioning, instability, and early implant loosening. Similarly, planning corrective osteotomies for distal radius malunion requires complex two-dimensional and three-dimensional measurements that are ideal for computational automation [4]. In the studies included in this review, automated pipelines for postoperative measurement often achieved intraclass correlation coefficients (ICCs) comparable to, or higher than, manual measurements, while substantially reducing analysis time, suggesting that computer vision can deliver quantifiable gains in both efficiency and measurement consistency.

In recent years, computer vision, a subfield of artificial intelligence (AI) concerned with teaching computers to interpret visual data, has transformed the analysis of medical imaging. Deep learning has enabled machines to detect fractures and classify pathologies with levels of accuracy that approach those of experienced radiologists [5,6]. In musculoskeletal imaging, early work has largely focused on trauma detection in long bones and degenerative disease grading in weight-bearing joints, such as the knee and hip. Upper limb applications, therefore, represent both a logical extension of this work and a clinically important test bed for translating these technical advances into everyday orthopaedic decision-making, particularly where current practice is constrained by diagnostic error and time-consuming, manual measurements.

In contrast, upper limb imaging presents unique challenges. The anatomy of the wrist and hand involves small, overlapping bones. The elbow is characterised by complex three-dimensional geometry and overlapping structures, making two-dimensional radiographic interpretation difficult. Furthermore, a significant portion of upper limb pathology involves soft tissues (e.g., rotator cuff tears and carpal tunnel syndrome), which require MRI or ultrasound. Datasets for these modalities are comparatively small, less standardised, and harder to label.

Despite these difficulties, advances in computer vision have begun to demonstrate potential in specific applications, such as automated wrist fracture detection and quantitative postoperative analysis following shoulder arthroplasty [7-9]. The upper limb is particularly well-suited to the development of computer vision tools because radiographs remain the first-line investigation for most conditions, and because the anatomical regions are relatively compact. Automated interpretation could, therefore, play a role in both emergency triage, where missed fractures are well documented, as well as in elective care, where precise measurement of joint morphology advises surgical planning and outcome assessment.

However, the true extent and maturity of computer vision research in upper limb orthopaedics remain unclear. This scoping review specifically aims to comprehensively map how computer vision has been applied across upper limb orthopaedic imaging. By outlining the landscape of published models, we seek to identify the predominant clinical and methodological trends, highlight gaps, and define the key priorities for translating these promising technical tools into validated, clinically useful applications, with particular emphasis on their potential to reduce diagnostic errors and improve the efficiency and reproducibility of surgical planning.

## Review

Methods

This review followed the PRISMA extension for scoping reviews (PRISMA-ScR) [10]. The PRISMA-ScR checklist and guidelines are publicly available for research use and did not require a license.

Eligibility Criteria

Studies were included if they analysed imaging of the upper limb (shoulder, elbow, wrist, or hand); used an automated or semi-automated computer vision method, such as segmentation, classification, landmark detection, measurement of radiographic parameters, three-dimensional reconstruction, or model-based interpretation; and used radiographs, CT, MRI, or ultrasound. Studies were excluded if they used only manual visual assessment without computational analysis; did not involve imaging; focused on non-musculoskeletal conditions; were animal studies; were not written in English; described imaging acquisition without any analytic algorithm; were conference abstracts without full-text availability; or were ongoing clinical trials. Systematic reviews were included because they summarised model performance and formed a relevant part of the evidence base.

Search Strategy

Ovid MEDLINE and Embase were searched (January 1995-October 2025) using terms combining upper limb anatomy, computer vision, or artificial intelligence. Search results were de-duplicated and exported for screening. The search string used was: (shoulder OR elbow OR wrist OR hand OR humerus OR radius OR ulna OR carpus OR metacarpal* OR phalanx OR "upper limb" OR "upper extremit*") AND (computer vision OR artificial intelligence OR machine learning OR deep learning OR neural network*) AND (imaging OR radiograph* OR X-ray OR CT OR "computed tomography" OR MRI OR "magnetic resonance" OR ultrasound).

Study Selection

Results were exported in a CSV format and manually reviewed. Duplicates were removed, and two reviewers screened titles and abstracts, and reviewed full texts. Disagreements were resolved with a senior reviewer.

Data Charting

Data was extracted into a structured chart, including study details, anatomical focus, imaging modality, computational method, dataset characteristics, and reported performance metrics.

Methodological Quality and Risk of Bias Assessment

Methodological quality was appraised using AI-specific adaptations of QUADAS-AI for diagnostic accuracy studies and PROBAST-AI for prediction and measurement models [11,12]. Each study was assessed across four domains: (1) dataset selection and representativeness, (2) model development and validation strategy, (3) reference standard generation, and (4) transparency of analysis and reporting. Judgements reflected study design (retrospective vs prospective), data provenance (single-centre vs multi-centre), validation approach (internal vs external testing), and markers of reproducibility, such as use of public datasets, inter-rater reliability reporting, and availability of code or model details. Two reviewers independently generated an overall risk-of-bias rating for each study, resolving discrepancies through consensus.

The tools applied (QUADAS-AI, PROBAST-AI, and the CLAIM checklist) are open-access frameworks designed for research use [11-13]. All are freely available for academic and non-commercial applications, and do not require additional licensing. These instruments were applied in accordance with their published methodological guidance.

Results

Study Selection

A total of 4,459 records were identified. After removal of 1,709 duplicates, 2,750 titles and abstracts were screened. Four hundred seventy full-text articles were reviewed, and 16 studies met the inclusion criteria (Table 1).

**Table 1:** Summary of included studies evaluating computer vision applications in upper limb orthopaedic imaging (n = 16). This table presents the key characteristics of all studies included in the upper-limb scoping review, detailing the article title, authors, publication year, journal title, imaging modality used, type of computational or deep learning model applied, and the risk of bias [11-29]. The included studies span radiographs, CT, and MRI modalities, and encompass a range of tasks including fracture detection and classification, segmentation, automated measurement, morphology assessment, and postoperative analysis.

Authors (Year)	Study Title	Imaging Modality	Primary Task	Algorithm/Approach	Risk of Bias (QUADAS-AI/PROBAST-AI Summary) [11-13]
Bouslimi et al. (2025) [14]	Enhanced diagnosis of paediatric wrist fractures using deep learning	CT, X-ray	Detection	Deep learning (YOLOv10 with AC-BiFPN, SimAM, WIoU loss)	Moderate - Large paediatric wrist dataset (GRAZPEDWRI-DX); strong internal performance; single-dataset evaluation with no multicentre external validation.
Rakesh and Akilandeswari (2022) [15]	Bone fracture detection using morphological and comparing the accuracy with genetic algorithm	CT	Detection, Segmentation	Morphological image processing and genetic algorithm	High - Very small wrist fracture sample (n = 32) from Kaggle; retrospective; unclear ground-truthing and validation procedures; high overfitting risk.
Min et al. (2023) [16]	Automatic classification of distal radius fracture using a two-stage ensemble deep learning framework	CT, X-ray	Classification, Detection	Two-stage ensemble: YOLOv5 region of interest (ROI) detection + EfficientNet-B3 classifier	High - Single-centre retrospective cohort; cross-validation only; no independent external test set; limited reporting on annotation reliability.
Yang et al. (2024) [17]	Artificial intelligence to automatically measure glenoid inclination, humeral alignment, and the lateralisation and distalisation shoulder angles on postoperative radiographs after reverse shoulder arthroplasty	CT, X-ray	Measurement, Registration, Segmentation	U-Net segmentation with an image-processing measurement pipeline	Moderate - Well-defined measurement endpoints with good observer agreement; retrospective single-centre series; no external or multicentre validation.
Ahmed et al. (2024) [18]	Enhancing wrist abnormality detection with YOLO: Analysis of state-of-the-art single-stage detection models	CT	Detection	Deep learning (YOLOv5/6/7/8 single-stage detectors)	Moderate - Large paediatric wrist dataset; comprehensive internal benchmarking; retrospective single-centre/single-dataset design without independent external validation.
Liu et al. (2024) [19]	Detection of clavicle fracture after martial arts training based on 3D segmentation and multi-perspective fusion	CT	3D reconstruction, Classification, Detection, Segmentation	3D segmentation with multi-perspective feature fusion (unspecified network)	High - Single institutional dataset; limited methodological detail on labelling and model training; no external testing or reproducibility reporting.
Li et al. (2025) [20]	Fracture detection of the distal radius using a deep-learning-based dual-channel feature fusion algorithm	X-ray (AP + Lateral)	Fracture detection	Dual-channel feature fusion model based on Faster R-CNN + ResNet-50 with deformable & separable attention; focal loss to address class imbalance	High - Single-centre retrospective dataset; exclusion of complex fractures; limited external validation; unclear reporting of annotation protocol and inter-rater reliability.
Javed et al. (2023) [21]	Wrist fracture prediction using transfer learning, a case study	CT, X-ray	Classification, Detection, Segmentation	ResNet-101 transfer learning (WFP-TL deep CNN)	High - Dataset origin and size not clearly specified; retrospective design; limited transparency on ground truth and validation; no external testing.
Kishor et al. (2025) [22]	Osteo fracture identification using deep learning techniques	CT, X-ray	Classification, Detection, Segmentation	Deep learning on MURA (CNN-based fracture vs non-fracture classification)	Moderate - Large public multi-bone dataset (MURA); internal evaluation only; orthopaedic sub-cohorts and labelling processes not fully described.
Smith et al. (2011) [23]	Volume-slicing of cone beam computed tomography images for navigation of percutaneous scaphoid fixation	CT, Fluoroscopy, X-ray	3D reconstruction, Measurement, Registration, Segmentation, Surgical planning	CBCT volume-slicing navigation with optical tracking and 3D segmentation	High - In vitro rapid-prototyped wrist model; no patient data; preclinical feasibility study with limited clinical generalisability.
Caiti et al. (2019) [24]	Implementation of a semiautomatic method to design patient-specific instruments for corrective osteotomy of the radius	CT	3D reconstruction, Measurement, Registration, Segmentation, Surgical planning	Semi-automatic CAD pipeline for patient-specific plate design	High - Method-development study with limited case numbers; no formal diagnostic or outcome validation; primarily technical feasibility.
Oonk et al. (2023) [25]	Quantification of the methodological error in the kinematic evaluation of the distal radioulnar joint (DRUJ) using dynamic CT	CT	3D reconstruction, Motion analysis, Registration, Segmentation	3D CT segmentation with rigid registration and centroid-based motion tracking	High - Single cadaver specimen; focus on methodological error rather than clinical performance; very limited external validity.
Muto et al. (2017) [26]	Development of a three-dimensional rotator cuff tendon magnetic resonance imaging system	CT, MRI	3D reconstruction, Classification, Measurement, Surgical planning	Manual tracing with 3D MRI reconstruction and medical image processing, analysis, and visualisation (MIPAV) processing	High - Small cohort; labour-intensive manual segmentation; no automated model or external validation; early technical feasibility.
Daluiski et al. (2017) [27]	A convolutional neural network automatically detects and localises fractures of the distal radius with greater accuracy than inexperienced clinicians	CT, X-ray	Detection, Segmentation	Custom CNN for semantic segmentation and heatmap-based fracture localisation	Moderate - Very large radiograph dataset with held-out test set and clinician comparison; single-centre; no multicentre external validation.
Beek et al. (2008) [28]	Validation of a new surgical procedure for percutaneous scaphoid fixation using intra-operative ultrasound	CT, Fluoroscopy, Ultrasound	Measurement, Registration, Surgical planning	CT-based preoperative planning with 3D ultrasound registration and optical tracking	High - Laboratory/phantom validation only; small number of trials; no clinical patient cohort or outcome data.
Qiu and He (2025) [29]	X-Yolo: A method for detecting wrist fractures in children based on dynamic feature enhancement and lightweight design	CT, X-ray	Detection, Measurement	YOLOv8-based lightweight detector (HGNetV2 backbone, DySample, GSConv)	High - Preprint using a single paediatric wrist dataset; promising internal performance but no peer-reviewed reporting or external validation.

**Figure 1:**
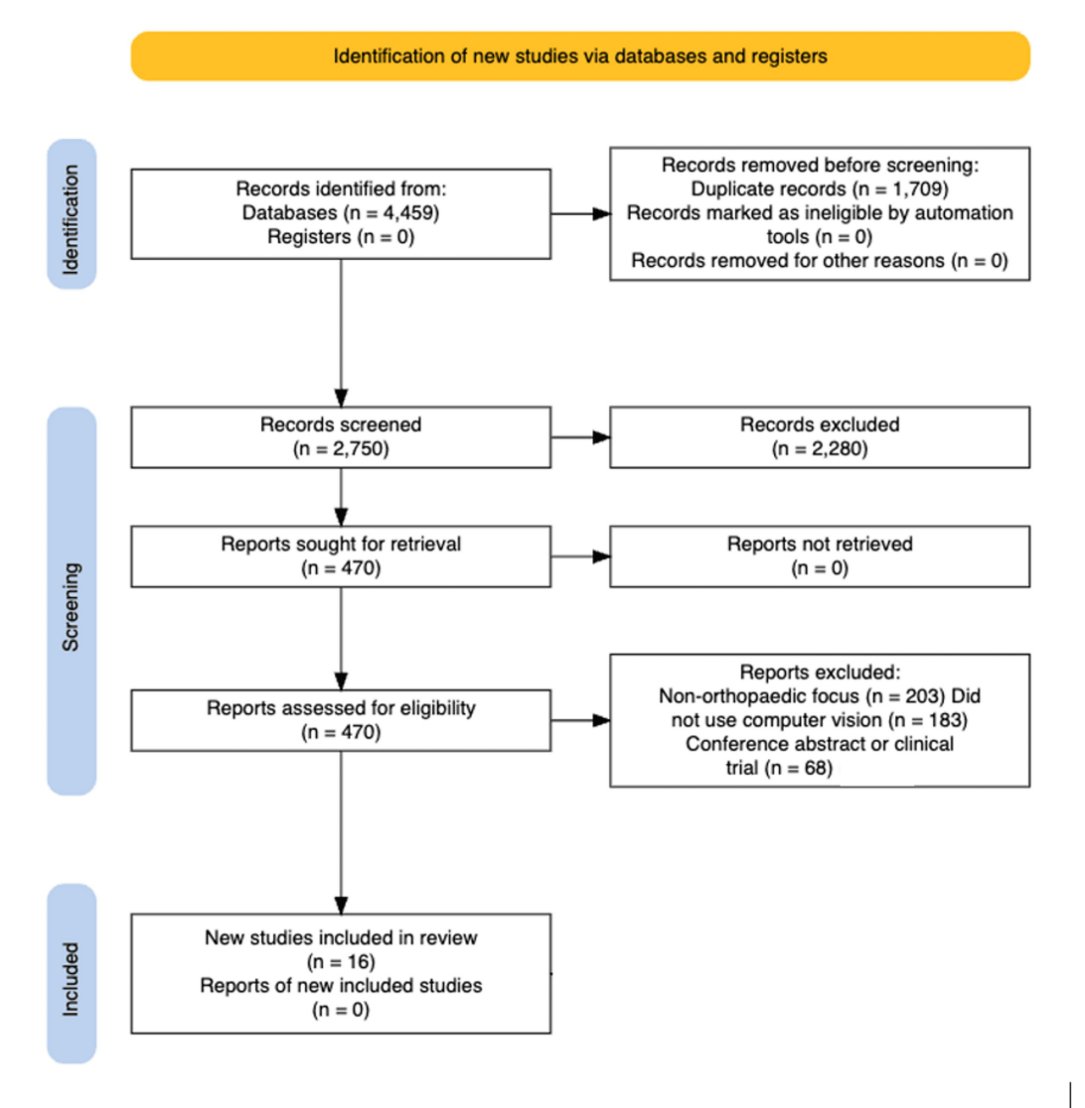
PRISMA flow diagram illustrating the study selection process. The PRISMA flow diagram [10] shows a total of 4,459 records were identified through database searches. After removal of 1,709 duplicates, 2,750 records were screened by title and abstract, with 2,280 excluded. Full texts were sought for 470 articles, all of which were retrieved. Following full-text assessment, 454 articles were excluded (203 were non-orthopaedic, 183 did not use computer vision methods, and 68 were conference abstracts or clinical trials). Sixteen studies met the predefined eligibility criteria and were included in the final review.

The PRISMA flow diagram is shown in Figure 1.

Study Characteristics

Most studies examined wrist (n = 8) or shoulder (n = 6) imaging. Only two studies investigated the elbow or hand. Radiographs were the most common modality (13/16), followed by CT (4/16), and MRI (2/16). A small number of studies combined multiple imaging types.

Tasks and Computational Approaches

The most frequent task was fracture detection or classification, particularly in wrist radiographs. Algorithms ranged from CNN classifiers and detection networks (e.g., YOLO) to ensemble models combining object localisation and classification stages. Several shoulder studies used segmentation-based approaches, most commonly U-Net architectures, to automate geometric measurement of postoperative parameters, such as glenoid inclination and humeral alignment. These deep learning models, including U-Net and YOLO, are standard, open-source architectures widely used in computer vision. A small number of studies used traditional computer vision techniques or statistical shape modelling for morphological analysis. While these approaches demonstrated reasonable accuracy on small datasets, their generalisability was limited.

Dataset Characteristics and Validation

Dataset sizes varied from fewer than 500 to more than 20,000 radiographs. Most were retrospective and single-centre in origin. Label generation was typically performed by orthopaedic specialists or radiologists. External validation was rare, and few studies described data governance, annotation protocols or inter-rater reliability testing.

Key Thematic Findings

The included studies highlight several key trends. Wrist fracture detection models, often based on CNN and YOLO, achieved strong diagnostic performance, with reported area under the ROC curve (AUROC) values above 0.9. Some of these models used attention-enhanced networks to reduce false negatives and improve speed, suggesting a clear potential for triage support in acute care. Two studies used systems to distinguish intra-articular from extra-articular distal radius fractures, which could help reduce unnecessary CT scanning. In the shoulder, segmentation pipelines successfully automated key geometric measurements after arthroplasty, with reported ICCs exceeding 0.9 compared with manual measurements, and significantly faster analysis times. Both AUROC and ICC are standard, non-proprietary statistical metrics for evaluating model performance. Older methods using contours or landmarks performed adequately on small datasets, but validation gaps were a consistent theme. Only two studies used an external test set, no prospective trials were identified, and reporting quality varied, limiting reproducibility.

Methodological Quality and Risk of Bias Findings

Analysis of the text showed that methodological quality was generally limited across the 16 studies (Table 1). Eleven studies were rated as overall high risk of bias, and five as moderate. Most were retrospective and single-centre, with small datasets, and minimal external validation. Selection bias was common, reflecting restricted populations, while model-development and validation procedures were often incompletely described. Reference standards were usually derived from a limited number of expert reviewers, without reported inter-rater reliability. Only two studies explicitly used independent test cohorts, and none were prospective. Although internal performance metrics were frequently high, the lack of external validation and transparent reporting reduced confidence in the generalisability of findings.

Discussion

This review identified 16 studies applying computer vision to upper limb orthopaedic imaging, confirming that this remains an emerging but expanding field. Although limited in volume compared with other areas of medical imaging, the included studies demonstrate clear technical progress and growing feasibility across specific diagnostic and measurement tasks.

Most work to date has focused on the wrist, reflecting both clinical need and technical suitability. Radiographs form the backbone of acute wrist fracture diagnosis, and their relatively simple two-dimensional structure lends itself well to convolutional analysis. Reported performances (particularly in paediatric cohorts) were high, indicating realistic potential for automated triage systems in emergency care. A reliable system that flags likely fractures for immediate review could shorten waiting times, reduce missed injuries, and support non-specialist staff during high-volume periods [30].

Shoulder-focused studies targeted a different problem: quantitative postoperative assessment. Automated segmentation and geometric modelling achieved excellent agreement with expert measurements for parameters such as glenoid inclination, version, and humeral offset. These tasks are time-consuming and operator-dependent when performed manually, yet are central to assessing implant positioning and arthroplasty outcomes [31]. Automating these measurements offers a clear path to routine quality assurance and more standardised reporting, with potential value for implant registries and comparative research.

Despite encouraging technical performance, the methodological appraisal highlights that current upper limb computer vision research remains early in maturity. Many studies relied on small, single-centre datasets, lacked transparent annotation methodology, and performed only limited validation. These design features create a substantial risk of bias and restrict generalisability. Multi-centre datasets, consensus-based labels, and external or prospective validation remain infrequent, yet are essential for establishing clinical credibility and aligning with frameworks such as QUADAS-AI, PROBAST-AI, and CLAIM [11-13].

A further limitation is the well-recognised problem of “domain shift”: models trained on a single institution’s radiographs often perform poorly when applied to images from other centres, or different imaging systems. As a result, reported accuracies may not reflect real-world performance. Shoulder studies additionally illustrate the persistent “metal-artifact problem,” where postoperative radiographs and CT scans are degraded by implants, complicating automated assessment of the bone-implant interface [32].

An equally important gap is the under-representation of soft-tissue imaging. The upper limb encompasses a large burden of tendon, ligament, and nerve pathology, particularly around the shoulder and wrist, but few studies addressed MRI or ultrasound interpretation [33]. High-impact soft-tissue targets for future computer vision work include automated detection and characterisation of rotator cuff tears, labral pathology, tendon and ligament injuries, and median nerve compression in carpal tunnel syndrome. This restricts current models largely to fracture detection and bony metrics, and means that these soft-tissue conditions currently remain underexplored by AI-based imaging tools, leaving major clinical questions unaddressed.

Another observation is the lack of workflow integration or evaluation of clinical impact. None of the included studies reported whether automated tools affected diagnostic accuracy, time-to-treatment, surgical planning decisions, or patient outcomes. High accuracy alone is insufficient; real-world utility must be demonstrated through prospective testing and meaningful clinical endpoints, such as reduced revision rates, or improvements in patient-reported outcomes.

This scoping review has limitations. Restricting the search to English-language publications and two databases may have excluded relevant work. Future research should now shift from proof-of-concept modelling toward clinically integrated systems. This will require larger, diverse, multi-centre datasets, potentially supported by privacy-preserving strategies such as federated learning, alongside deliberate efforts to address the underdeveloped area of soft-tissue imaging through expanded MRI and ultrasound datasets, focused on conditions such as rotator cuff pathology, labral and tendon injuries, and carpal tunnel syndrome. At the same time, the field must progress beyond simple detection tasks toward prognostic tools that assist in treatment selection and risk stratification. Finally, successful implementation will depend on practical considerations such as seamless radiological software integration, cost-effectiveness, regulatory approval, and clear frameworks for professional responsibility. This will ensure these technologies can be adopted safely and meaningfully within routine clinical workflows.

Overall, the evidence indicates that upper limb orthopaedic imaging is at the early stages of a significant transition. With thorough validation and close collaboration between clinicians, engineers, and healthcare systems, computer vision has the potential to support both acute and elective upper limb care and meaningfully enhance diagnostic and surgical practice.

## Conclusions

Computer vision is increasingly being applied to upper limb orthopaedic imaging, though the field remains in its early stages. The 16 included studies demonstrate that automated fracture detection and radiographic measurement are technically achievable, with strong accuracy and reliability. Most developed applications lie in wrist fracture detection and postoperative shoulder analysis. Despite these advances, widespread clinical implementation is limited by small sample sizes, retrospective design, and lack of prospective testing.

To progress from technical feasibility to clinical application, future studies must prioritise external validation and transparent reporting. Expanding research into MRI- and ultrasound-based models may also unlock potential for soft-tissue and intra-operative applications. Ultimately, computer vision has the capacity to become a valuable adjunct to orthopaedic imaging practice, improving diagnostic consistency and efficiency. However, real progress will depend on collaboration across clinical, engineering, and regulatory domains, to ensure that these technologies are safe, reliable, and ethically beneficial to patients.
